# Harnessing haloarchaea from halophyte *Atriplex nummularia* rhizosphere to enhance salt stress tolerance in maize seedlings

**DOI:** 10.1186/s40793-025-00698-2

**Published:** 2025-12-11

**Authors:** João Paulo Ventura, Gileno Vieira Lacerda-Júnior, Theopi Rados, Alex Bisson, Paulo Ivan Fernandes-Júnior, Itamar Soares Melo

**Affiliations:** 1 College of Agriculture “Luiz de Queiroz”, University of São Paulo, Piracicaba, SP Brazil; 2 Embrapa Environmental, Jaguariúna, SP Brazil; 3 Department of Biology, Brandeis University, Waltham, MA 02454 USA; 4 Embrapa Semiarid, Petrolina, PE Brazil

**Keywords:** Archaea, Plant-microbiome, Salinity, *Atriplex nummularia*, *Haladaptatus*

## Abstract

Soil salinization is a critical global issue threatening agricultural productivity and significantly reducing the availability of arable land. Effective mitigation and recovery strategies are vital for sustaining food production, especially in the context of climate change. Halophytic plants, such as *Atriplex nummularia*, have shown potential for remediating saline soils, though their large-scale application remains limited. An alternative approach involves leveraging microorganisms adapted to saline environments to enhance plant stress tolerance. In this study, we investigated the microbiome of *A. nummularia* under saline and non-saline irrigation conditions to identify extremophilic microorganisms that promote salt stress tolerance. Through 16S rRNA analysis, we identified members of the genus *Haladaptatus* exclusively in the rhizosphere of salt-irrigated plants. These microorganisms were isolated and inoculated into maize crop systems to evaluate their ability to confer salt tolerance. Our results demonstrate that *Haladaptatus* strains significantly enhance salinity tolerance in maize, with a marked increase in the relative abundance of archaeal 16S rRNA in soils as NaCl irrigation levels rise. This study provides the first evidence that *Haladaptatus*, an archaeon isolated from the rhizosphere of a halophyte, can significantly enhance salt tolerance in an agriculturally important crop. These findings suggest a promising biotechnological application for improving crop resilience in saline environments, offering a sustainable strategy for addressing soil salinization and securing food production in the context of global climate challenges.

**Supplementary Information:**

The online version contains supplementary material available at 10.1186/s40793-025-00698-2.

## Introduction

Soil salinization has emerger as a problematic abiotic stressor in woldwide, leading to a decline in global crop productivity, promoting desertification, and reducing arable land [1–4]. From 1986 to 2016, soil salinity increased by 100 million hectares globally and continues to expand, driven by the intensification of agricultural and fertilizer practices [5, 6]. This scenario is particularly alarming in regions where agriculture is the cornerstone of economic stability and food security [7].

Salt accumulation significantly impacts glycophytic plants, such as maize, by increasing sodium and chloride concentrations in the soil. This leads to substantial agricultural production losses, primarily due to ionic and osmotic stress caused by soil salinization [8–11]. Conversely, halophytic plants such as *A. nummularia* require a specific salt concentration in the soil for optimal development. They possess potent mechanisms for the phytoremediation of saline areas, absorbing salts directly from the rhizosphere and accumulating them in their leaves [12]. Moreover, the strategic use of halophytes in bioremediation projects has been highlighted as a viable approach for reclaiming saline soils, thereby mitigating the environmental and economic impacts of soil salinization [13].

In response to environmental stresses, plants establish beneficial relationships with microbes, hosting a complex associated community. This community has the potential to enhance plant growth and improve mechanisms such as nutrient and mineral uptake, nitrogen fixation, and phytohormone production. Additionally, these microorganisms play a key role in protecting plants against pathogens and significantly bolstering the well-being and salt tolerance of halophytes [14–16]. Therefore, while some halotolerant and halophilic microorganisms, including *Bacillus*, *Halobacillus*, *Halomonas*, and *Salinibacter*, have been widely explored for their ability to promote salt stress tolerance in plants, the potential of archaea in interacting with plants and protecting them against biotic and abiotic stress remains underinvestigated. Specifically, the interaction between plants and haloarchaea warrants further exploration [17–19].

This microbial group thrives under extreme conditions characterized by high salt concentrations and elevated temperatures [20]. Their unique metabolic pathways and stress response mechanisms offer a rich source for biotechnological applications, especially in developing bioinoculants for saline agriculture [21]. These microorganisms, belonging to the archaea domain, possess mechanisms for regulating the intracellular concentration of Na + and Cl^-^ ions, enabling them to flourish in environments with salt concentrations exceeding 25% [11, 22, 23].

Recent studies suggest that Archaea should be recognized as an essential component of microbiome analysis, given their beneficial potential in interacting with their host and their involvement in plant health and nutrient cycling [18, 24]. Additionally, due to the adaptation mechanisms, halophilic archaea represent a promising biotechnology source of novel enzymes and other bioactive compounds that operate under stress conditions, including varying temperatures, high salt concentrations, and pH levels, which pose limitations for other microorganisms [25, 26].

The potential of archaea in assisting plants has recently garnered attention through unveiling their role in inducing resistance against both abiotic and biotic stress factors, such as cobalt exposure, salinity, and water restriction. However, the limited number of studies hinders a comprehensive understanding of the potential of archaea in agricultural systems [27–29]. Consequently, these findings suggest a broader application of archaea in agriculture, expanding beyond the mitigation of salt stress to address challenges associated with climate change. This opens a new frontier in exploring the role of archaea in plant–microbe interactions, stress mitigation, and environmental bioremediation, which could be pivotal for developing sustainable agricultural practices in saline and metal-contaminated soils.

However, the complex mechanism involved in the interaction between plants and archaea remain enigmatic, primarily due to the challenges associated with cultivating and deploying these microorganisms in agriculture [30]. Despite these constraints, archaea have surfaced as a novel strategy to navigate extreme conditions and enhance sustainable agriculture [31].

We hypothesize that *A. nummularia*, when subjected to saline irrigation, selects for microorganisms in its rhizosphere that are highly adapted to environmental salt fluctuations. These selected microorganisms, particularly halophilic archaea, hold significant potential as bioinoculants for inducing salt tolerance in agricultural crops. By exploring the impact of saline conditions on the rhizosphere microbiome of *Atriplex*. Our study aims to identify these specialized archaea and evaluate their ability to induce salt tolerance in agricultural crops. This approach highlights the role of halophytic plants as reservoirs of stress-adapted microbiomes and underscores their potential application in sustainable agriculture through microbiome-based strategies.

## Methodology

### Soil sampling and area characterization

Soil samples were collected from two distinct sites (CEC and SNT) located in Petrolina, Pernambuco State, Brazil, within the Caatinga Biome, a semi-arid region. The CEC site, situated at 9°04′04.6"S 40°19′04.0"W, represents an agricultural field where *A. nummularia* has been cultivated under saline irrigation. In contrast, the SNT site, located at 9°03′13.4"S 40°17′51.4"W, is an agricultural field where the plants were grown without saline irrigation.

At each site, soil samples were collected in triplicate from three distinct rhizosphere points, along with one bulk soil sample also collected in triplicate from each field, resulting in a total of 24 samples (CEC: 9 rhizosphere samples and 3 bulk soil samples; SNT: 9 rhizosphere samples and 3 bulk soil samples). The samples were immediately placed in sterile containers to prevent contamination and maintained under refrigeration during transport to the Brazilian Agricultural Research Corporation (Embrapa), located in Jaguariúna, São Paulo State, Brazil. The soil samples were subjected to physicochemical analyses, including measurements of pH, electrical conductivity, and sodium concentrations, which provided essential data to confirm the salinity differences between the two sites (Table S1).

### Bacterial and archaea communities assessment using 16S rRNA amplicon sequencing

The genomic DNA from the microbial community of rhizosphere soils and bulk soils was extracted using the PowerSoil DNA extraction kit (MoBio Laboratories Inc.) following the manufacturer's instructions. The concentration and purity were estimated using a NanoDrop®ND-2000 Spectrophotometer (Thermo Scientific, Wilmington, DE, USA). The integrity of the extracted material was confirmed by 1% agarose gel electrophoresis in TAE buffer (1X). The PCR reaction for the amplification of the 16S rRNA gene was conducted with a final volume of 25 μL, consisting of 13 μL of DNase-free ultrapure water, 10 μL Phusion master mix (final concentration 1X), 0.5 μL of primer 515 FB (5′- GTGYCAGCMGCCGCGGTAA-3′) (final concentration of 0.2 μM), 0.5 μL of primer 806RB (5′-GGACTACNVGGGTWTCTAAT-3′) (final concentration of 0.2 μM), and 1 μL of genomic DNA (final concentration of 5 ng) [32]. Amplification was conducted in a Veriti thermal cycler (Applied Biosystems, USA) under the following amplification conditions: 94 °C for 5 min, followed by 30 cycles of 94 °C for 30 s, 57 °C for 45 s, 72 °C for 1 min, and final extension of 72 °C for 10 min. The size and specificity of the fragments obtained were verified by 1.5% agarose gel electrophoresis, and Illumina adapters were ligated in a PCR reaction (index Nextera XT Index Primer 1 (N7xx) and Nextera XT Index Primer 2 (S5xx)). The purification of PCR products was performed using AMPure XP Beads (Beckman Coulter, Life Sciences). Library quantification was performed with the KAPA Library Quantification kit for Illumina (Roche), and sequencing was performed on Illumina MiSeq equipment in 2 × 250 bp runs.

### Bioinformatics and data analysis

The raw data were processed using Dada2 version 1.21.0 [33]. The primers were removed using Cutadapt version 3.4. Quality control was performed and reads of low quality (Q20 or lower) were discarded. To generate ASVs, chimera sequences were removed using the"removechimera"command in the DADA2 package. After data processing, the taxonomic classification was performed using the"AssignTaxonomy"function of the DADA2 package against the SILVA 138 v1.2 database. Sequences assigned as chloroplast or mitochondrial DNA were removed from the ASV table.

Data analyses were conducted in the R environment using the"vegan"and"phyloseq"packages unless otherwise stated. All analyses were based on rarefied data from the sample with the lowest coverage (25,000 reads) using the “rrarefy” function. Alpha and beta diversity metrics were used to assess the effect of saline irrigation on archaeal composition and diversity [34, 35]. PCoA analyses were performed using the Bray–Curtis dissimilarity metric based on the abundance table. The Differentially enriched microbial groups were detected using Microbiome Process package, and the differential statistical results were acessed using the Kruskal Wallis test [36]. The PERMANOVA was used to determine differences between treatments and collection points and ASVs affiliated with the Archaea domain were filtered for subsequent functional prediction analysis using Tax4 Fun2 [37]. Plots were generated using ggplot2 and microbial process package, and statistical comparisons regarding functional prediction were performed using the STAMP program [38].

### The isolation of haloarchaea strains

The isolation of halophilic archaea was performed using the enrichment of 1 g of rhizospheric soil collected from *A. nummularia* plants at the CEC field. The soil sample was added to a 250 mL Erlenmeyer flask containing 50 mL of modified CMD medium (10 g/L glucose, 10 g/L sucrose, 1 g/L cellulose, 1 mL of pyruvic acid solution (1:1), pH 7.5) [39]. Considering that the Caatinga biome is characterized by semi-arid conditions and high temperatures, the incubation temperature of 40 °C was chosen to simulate the natural environment of the microbial community and to select halophilic archaea capable of thriving under such conditions. The soil was incubated at 40 °C with shaking at 180 rpm, and enrichment samples were collected at 15-day intervals. These samples were inoculated onto Petri dishes containing solid CMD medium, which were subsequently incubated until visible colonies appeared. The resulting colonies were purified and cryopreserved at −80 °C in a solution containing 20 mL of 30% saltwater (NaCl 240 g/L, MgCl₂·6H₂O 30 g/L, MgSO₄·7H₂O 35 g/L, KCl 7 g/L, 1 M Tris–HCl (pH 7.5) 5 mL) and 80 mL of glycerol PA [39]. The archaeal strains were deposited in the Culture Collection of Microorganisms of Agricultural and Environmental Importance (CMAA) of the Brazilian Agricultural Research Corporation—Embrapa, Jaguariúna, São Paulo State, Brazil.

### Identification of isolates using 16S rRNA gene sequence

The isolated strains were cultured in a CMD broth medium, and genomic DNA was extracted using a Wizard® Genomic DNA Extraction kit (Promega, USA) according to the manufacturer's instructions. The 16S rRNA gene sequence was amplified using the universal Archaea primers ARC 8f/ARC 1492r [40]. The PCR product was then purified using the Wizard® SV Gel and PCR Clean-Up System (Promega, USA). Sequencing was performed on an automated ABI Prism 3730 instrument using the BigDye Terminator Cycle Sequencing Kit (Thermo Fisher Scientific, Waltham, MA, USA) with the following primers: ARC 519r (GGTDTTACCGCGGCKGCTG), ARC 915r (GTGCTCCCCCGCCAATTCCT), ARC 1492r (GGCTACCTTGTTACGACTT), ARC 21f (TTCCGGTTGATCCYGCCGGA), ARC 344f (AYGGGGYGCASCAGGSG), and ARC 8f (TCCGGTTGATCCTGCC). The assembly and quality control of the sequences were performed using the software CodonCode Aligner Version 1.6. The partial or full-length 16S rRNA sequences were compared with those of reference strains in the NCBI [42] and EzBioCloud [41] databases for taxonomic classification. In addition, phylogenetic trees based on the full-length 16S rRNA gene sequences of type strains most closely related to the isolates were constructed using MEGA 11 software [43] with the neighbor-joining based on the ClustalW alignment with default parameters [44, 45]. Bootstrap values were calculated using 1000 replications.

### Evaluation of potential halophilic archaea strain in mitigating salt stress in maize

The archaeal strains were cultivated in liquid CMD medium with a modified NaCl concentration (110 g/L NaCl, 20 g/L MgCl₂·6H₂O, 15 g/L MgSO₄·7H₂O, and 5 g/L KCl). After growth, the broth containing the cell culture was centrifuged for 5 min at 10,000 g, and the cells were resuspended in a saline solution containing 0.85% NaCl (v/w) at a concentration of 10⁸ CFU (OD600 nm), which was adjusted using the Shimadzu spectrophotometer. To assess cell viability, 100 μL of the suspension was inoculated onto solid CMD medium after exposure to the saline solution for 1 h and subsequently quantified.

Initially, an experiment was conducted using maize seeds in Murashige & Skoog (MS) medium with and without the addition of 200 mM NaCl to assess the influence of archaeal inoculation on seed germination. For this, nine hybrid maize seeds (variety BM709PRO02, Biomatrix^Ⓡ^) were treated with halophilic archaeal cells.

**Fig. 1:**
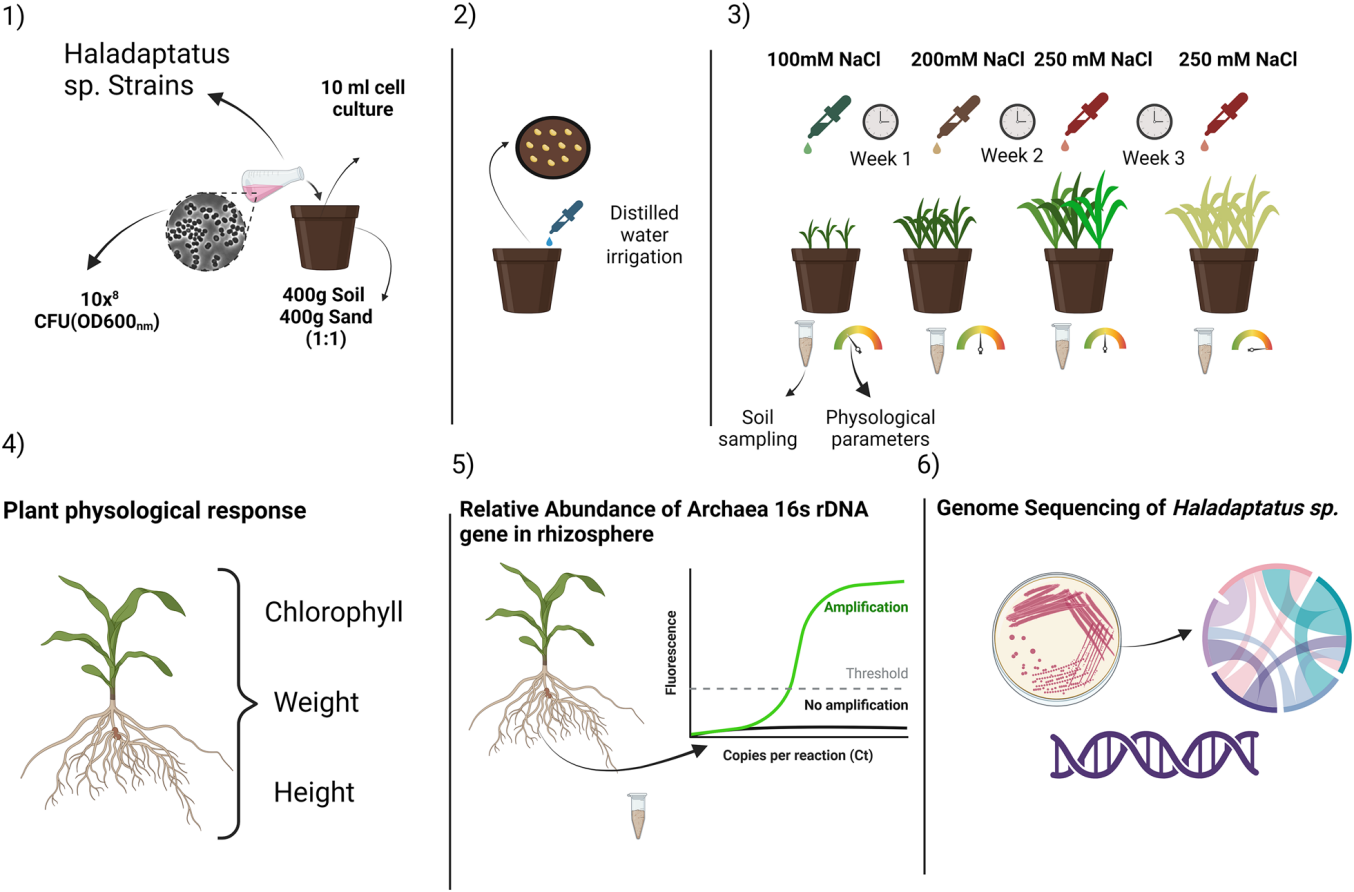
Greenhouse experimental workflow. The soil were previous treated with 10 mL archael strains at a concentration of 10^8^ CFU/mL (colony-forming units). Subsequently, 10 seeds were planted per pot. Physiological response were evalueted throughout the experiment, and soil samples were collected at the conclusion to measure the abundance of 16S rDNA. To undestand the archaea potential in alleviating salt stress in maize plants, the genome of two strains were sequenced and the functional and metabolic pathways were annotated

Following this initial test, a greenhouse experiment was conducted using maize plants. For this, 10 mL of each archaeal inoculum was applied to the soil before seed planting. Three days after the initial application, ten hybrid maize seeds (variety BM709PRO02, Biomatrix^Ⓡ^) were planted per pot. The experiment was conducted under controlled conditions: a constant temperature of 25 °C, relative humidity of approximately 80%, and a 12-h photoperiod using the EL011—EletroLab^Ⓡ^ system. The tests were performed in 1-L pots containing a substrate mixture of oxisol and sand in a 1:1 ratio (Table S2). A completely randomized design was employed, with five replicates per treatment. The treatments consisted of six strains of halophilic archaea, along with two control groups: one without archaea or salt and another without archaea but with salt (Fig. 1). Soil characteristics were analyzed at the Brazilian Agricultural Research Corporation (Embrapa) in Jaguariúna, São Paulo, Brazil.

Daily irrigation was performed using sterilized distilled water. An automated irrigation system was programmed to dispense 20 mL every 8 h (60 mL/day). After seed emergence (7 days after planting), seven seedlings were thinned, leaving only three seedlings per pot. Saline irrigation was then initiated according to the following schedule: 7 days with a 100 mM NaCl solution, followed by 7 days with a 200 mM NaCl solution, and finally 7 days with a 250 mM NaCl solution. Continuous irrigation with a 250 mM NaCl solution was maintained until symptoms appeared in plants that did not receive archaeal inoculation under the same saline conditions.

Chlorophyll levels were measured using the SPAD 502 Plus Chlorophyll Meter (Konica Minolta), and rhizosphere soil samples were collected at each salt concentration change during irrigation. These samples were stored in an ultra-freezer (− 80 °C) for subsequent analysis of the archaeal 16S rRNA gene.

The effects of inoculation on plant responses were evaluated by measuring fresh and dry shoot and root weight, relative water content, and the salt tolerance index. Statistical analyses, including ANOVA with Tukey’s post-hoc test, were performed to determine differences between treatments.

### Investigating the dynamics of archaea in the maize rhizosphere during salt stress mitigation

The copy number of the archaean 16S rRNA gene was quantified using quantitative PCR (qPCR) to assess the population dynamics of archaeal communities in the maize rhizosphere throughout the experiment. Rhizospheric DNA was isolated from samples and diluted to a final concentration of 5 ng to ensure assay standardization.

Amplifications were performed in triplicate using SYBR Green on a Step One Plus instrument (Applied Biosystems) and white-walled 48-well plates. The qPCR reactions were performed using archaea primers (ARC 344 F/ARC 915R). The amplification program consisted of an initial denaturation step at 95 °C for 5 min, followed by 40 amplification cycles of 1 min at 95 °C, 1 min at 60 °C, and 1 min at 72 °C, and a final elongation step of 15 s at 95 °C and 1 min at 60 °C for melting curve analysis. The standard curve was calculated using a pool of all samples with concentrations of 50 ng, followed by dilution to 10^7^ using 5 replicates for each concentration.

### Genome sequencing of *Haladaptatus* strains

Genome sequencing was performed using a hybrid approach combining Nanopore and Illumina sequencing technologies. Quality control and adapter trimming were performed using bcl-convert version 3.9.3 for Illumina sequencing and porkchop version 0.2.3_seqan2.1.1 for ONT sequencing. The hybrid assembly of Illumina and ONT reads was performed using Unicycler version 0.4.8, and assembly statistics were recorded using QUAST version 5.0.2. Assembly annotation was performed using Prokka version 1.14.5 and RAST version 2.0. The quality of the genome assembly was checked using BUSCO. Phylogenetic annotation was performed using FastANI. The genome circular view were creted using circus from web application https://www.bv-brc.org/. Pairwise sequence similarities were calculated using Type Strain Genome Server—TYGS. Phylogenies were inferred by the GGDC web server available at http://ggdc.dsmz.de/ using the DSMZ phylogenomics pipeline adapted to single genes [46]. Plant-Growth Promotion Genes (PGPG) traits were annotated using the *PGPg_finder* v1.1.0 [47].

## Results

### The effect of salinity on the rhizosphere microbiome of *A. nummularia*

**Fig. 2:**
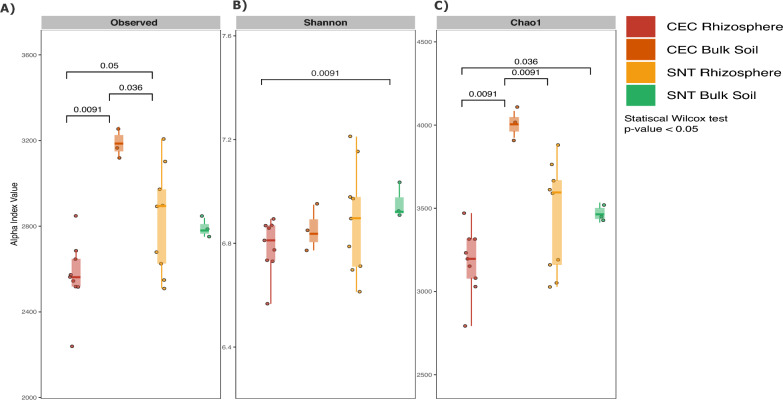
The diversity and richness of microbial communities compared between samples under saline and non-saline conditions. **A** Observed richness; **B** Shannon diversity index; **C** Chao1 richness index. Error bars represent the standard deviation. Significant differences between treatments were determined using the Wilcoxon test (*P < *0.05)

A total of 832,221 reads were recovered, with an average coverage of approximately 30,000 reads per sample. After removing ASVs affiliated with unidentified phyla, mitochondria, and chloroplasts, 5696 ASVs remained. The samples were normalized and rarefied to 25,377 reads per sample. Alpha and beta diversity analyses were then conducted to assess the effects of saline irrigation on the rhizosphere microbial community. Statistical analysis revealed significant differences in the number of observed ASVs (Fig. 2A). However, a significant difference in the Shannon diversity index was only observed when comparing the CEC rhizosphere to the SNT bulk soil, with no significant differences detected among other treatments (Fig. 2B). Additionally, significant differences in the Chao1 richness index were observed between the experimental fields CEC and SNT (ONE-WAY ANOVA, *p < *0.05), particularly among rhizosphere samples (*p < *0.01).

**Fig. 3:**
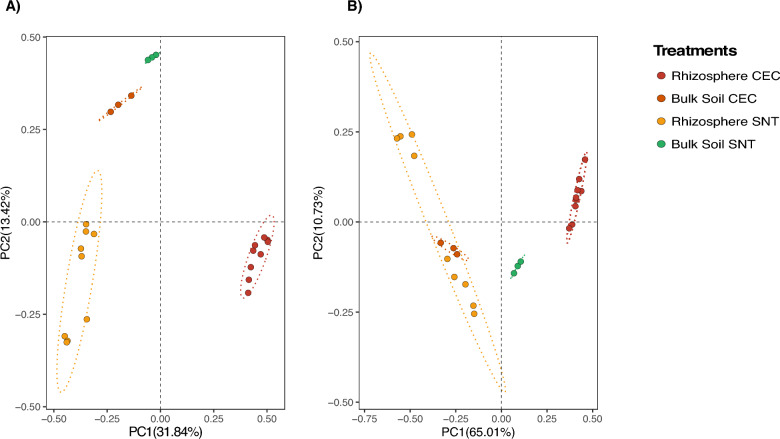
PCoA of Beta diversity in the microbiome using the Bray–Curtis distance. **A** The index was calculated for all ASVs. **B** The index calculated only for archaeal ASVs

Beta diversity analyses indicated a strong influence of salinity on the rhizosphere microbiome of *A. nummularia*. Principal Coordinate Analysis (PCoA) revealed a clear separation between soil and rhizosphere samples from the two experimental sites, highlighting the significant role of saline concentration in shaping microbial community structure (*P < *0.001) (Fig. 3A). The first principal coordinate (PC1) explained 31.84% of the variation in Fig. 3A and 65.01% in Fig. 3B, indicating a strong differentiation among treatments, while PC2 accounted for 13.42% and 10.73%, respectively, reinforcing the observed clustering pattern.

Notably, while alpha diversity metrics (Fig. 2) demonstrated significant differences in richness and evenness between experimental fields, beta diversity further emphasized the extent of community dissimilarity driven by salinity. The high dissimilarity observed in the CEC rhizosphere, in contrast to the bulk soil from the same site, underscores the cumulative effect of salt accumulation in shaping the microbial composition within the rhizosphere of *A. nummularia*.

Intriguingly, we conducted beta diversity analysis separately, focusing solely on sequences affiliated with the Archaea domain. Principal Component Analysis (PCoA) also unveiled a significant influence of saline irrigation on the archaeal community in the rhizosphere samples from the CEC field, as indicated by pronounced dissimilarity (Fig. 3B). These findings suggest that enriched archaeal groups may play a role in processes related to soil salts and, consequently, contribute to stress mitigation in *A. nummularia* plants.

### Microbial composition and enrichment of halophilic archaea in the rhizosphere of *A. nummularia* under saline irrigation

**Fig. 4:**
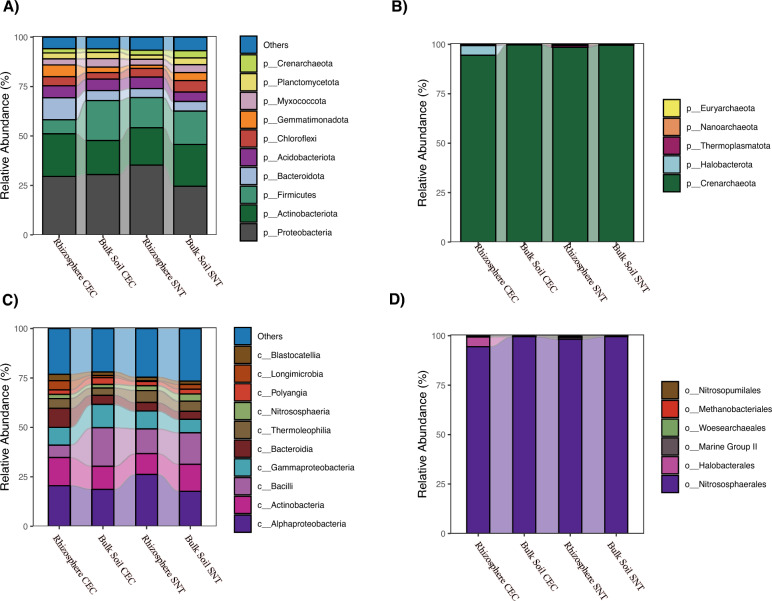
Relative abundance of microbiome composition among the sites. **A** Relative abundance of phylum level; **B** Relative abundance of phylum level only for *Archaea* ASVs; **C** Relative abundance at the class level showing the different composition among the sites; **D** Relative abundance only for archaea classified by order level

To assess variations in microbial composition across samples, we conducted comparative analyses on the relative abundance of the most enriched microbial groups. In both high- and low-salinity samples, the phyla *Proteobacteria (Pseudomonadota)* and *Actinobacteriota* were dominant (Fig. 4). However, notable compositional differences were observed between the samples. The abundance of the Firmicutes phylum decreased in the rhizosphere under saline irrigation, in contrast to its higher abundance in the CEC bulk soil. *Proteobacteria (Pseudomonadota)* and *Actinobacteriota* emerged as the bacterial phyla with the highest relative abundance (Fig. 4A).

Regarding Archaea, the phylum *Crenarchaeota* was present across all samples, but interestingly, the *Halobacteriota* phylum was only found in the rhizosphere samples from the CEC field (Fig. 4B). At lower taxonomic levels, we observed a slight reduction in the *Bacilli* class in saline-irrigated samples. This reduction was particularly evident when comparing the CEC bulk soil samples to the rhizosphere samples, a trend not observed in the SNT field samples (Fig. 4C). These subtle differences were further highlighted at the ASV level within the Archaea orders, where *Halobacteriales* was observed exclusively in these samples (Fig. 4D).

At the genus level, *Candidatus Nitrocosmicus* was the most abundant genus in both soil and rhizosphere samples (Supplementary Figure S1). In the CEC field, ASVs related to halophilic archaea were predominant, while in the SNT field, ASVs associated with ammonia-oxidizing archaea were more abundant. Notably, halophilic archaea were exclusively found in the rhizosphere samples from the CEC field, suggesting a potential synergistic effect, with particular prominence of the genus *Haladaptatus*, the most abundant among halophilic archaea.

**Fig. 5:**
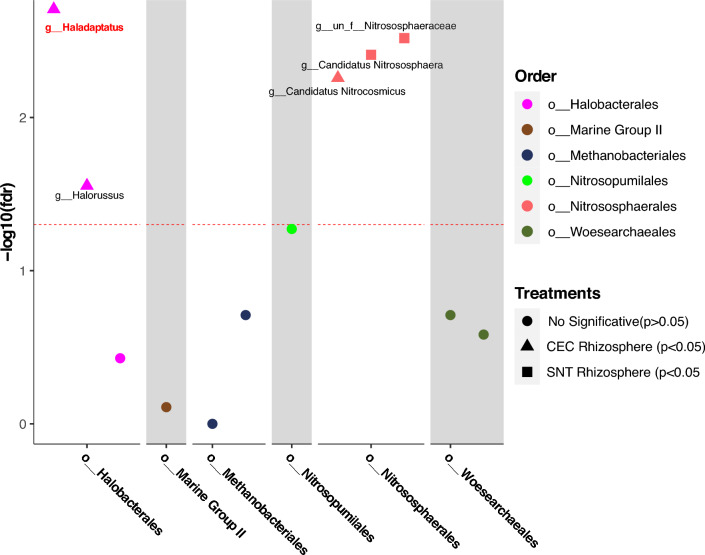
Differential enrichment group in Archaea ASVs using Kruskal Wallis with *p < *0.05, highlighting the *Haladaptatus* genus (in red) with high significative enrichment in the CEC Rizhosphere

Nevertheless, differential abundance analyses revealed a significant enrichment (*p < *0.05) exclusively in halophilic archaeal groups belonging to the genus *Haladaptatus* in salt-irrigated field (CEC), correlating with the direct accumulation of salts in the rhizosphere *of A. nummularia* (Fig. 5). Functional prediction analyses were conducted on ASVs belonging to the Archaea domain to explore potential functional attributes that halophilic groups associated with the rhizosphere of *A. nummularia* might utilize in mitigating salt stress.

## Functional diversity of archaeal communities in rhizosphere of *A. numularia* under salt stress

**Fig. 6:**
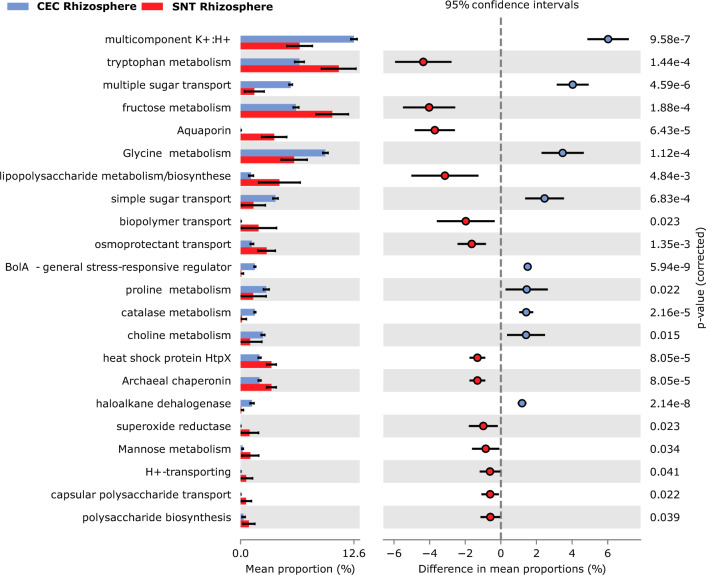
Functional prediction using Tax4fun2 comparing CEC and SNT rhizosphere samples. The functional analyses showed the significant statistical differences (*p < *0.05)

Using Tax4fun2, we conducted a prediction of the potential functional attributes within the archaeal communities of the *A. nummularia* rhizosphere, spotlighting crucial functions for salt stress tolerance. Intriguingly, cluster analyses unveiled a notable divergence in functional profiles between the samples from CEC and SNT fields. The rhizosphere archaeal community from the CEC field exhibited enriched functionality associated with sugar transport mechanisms, synthesis of protective osmolytes (e.g., glycine, proline, and choline), and the K + :H + multicomponet. In contrast, the SNT field rhizosphere revealed a pronounced abundance of functions related to polysaccharide metabolism, chaperones, and aquaporins (*P < *0.05) (Fig. 6). These findings illuminate the diverse functional strategies employed by halophilic archaea in the rhizosphere of salt-accumulating sites, potentially acting as a mitigative force against the detrimental effects of salt stress on *A. nummularia* plants.

## Isolation and identification of halophilic archaeal strain

To delve into the functional role of the rhizosphere community and recognizing the potential of the genus *Haladaptatus* in processes potentially related to the osmotic regulation of *A. nummularia*, we implemented a meticulous strategy unfolded to enrich rhizosphere soils from the field, with the goal of isolating of strains belong to this microbial group. The crafted enrichment strategy aimed to isolate halophilic archaeal strains in a culture medium, resulting in the successful isolation of six strains with robust growth potential across a spectrum of salt concentrations (6 to 30%, w:v) and temperatures (30 to 50 °C).

**Fig. 7:**
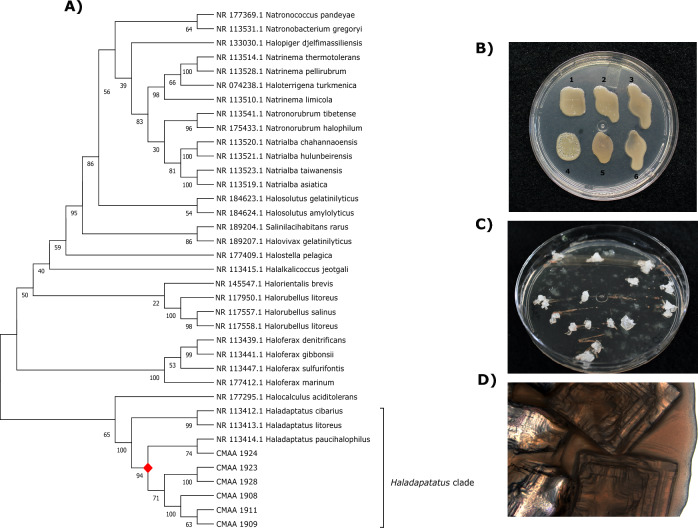
**A** Phylogenetic tree based on 16S rRNA gene sequences of Archaea, constructed by aligning sequences using Clustal W and the neighbor-joining method with 1000 bootstrap replicates. **B**) Strains used in the experiment on modified CMD culture medium. The numbers represent the following strains: 1—CMAA 1924; 2—CMAA 1923; 3—CMAA 1908; 4—CMAA 1911; 5—CMAA 1928; 6—CMAA 1909. Note the presence of crystal formation due to salt accumulation, possibly within the biofilm. **C**) Growth of strain CMAA 1911 in an isolated plant with accumulated salt. **D**) Salt crystals forming at the growth site of strain CMAA 1911

Taxonomic affiliation was determined through sequencing the 16S rRNA gene, revealing spanning from 93.61 to 97.89% with the species *Haladaptatus paucihalophilus* (Table S3). The phylogenetic tree, constructed from sequences most closely related, delineates the evolutionary relationships of genus *Haladaptatus* (Fig. 7A). Strains CMAA 1908, CMAA 1924, CMAA 1911, CMAA 1909, CMAA 1928, and CMAA 1923 coalesced into a distinct clade, hinting at a possible affiliation as a new species. These strains exhibit similar morphological growth in the medium with a range of 10 to 27% NaCl concentration (Fig. 7B). Interestingly, some salt crystals were forming during the growth of certain strains (Fig. 7C–D).

## Effects of soil inoculation with Haloadaptatus strains on maize seedlings under saline conditions

**Fig. 8:**
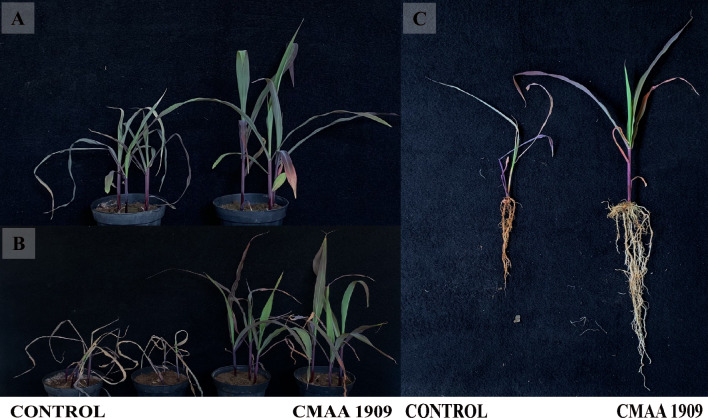
Maize response to *Haladaptatus* sp. inoculation under salt stress conditions. **A** Initial visible symptoms due to salt irrigation (200 mM NaCl) in maize plants inoculated with CMAA 1909. **B** After 7 days of 250 mM NaCl irrigation, non-inoculated plants have exhibited severe symptoms compared to the inoculated plants. **C** Effect of salt irrigation at the end of the experiment after 7 days under 250 mM NaCl; the roots of inoculated plants show improved protection against salt irrigation compared to the control

**Fig. 9:**
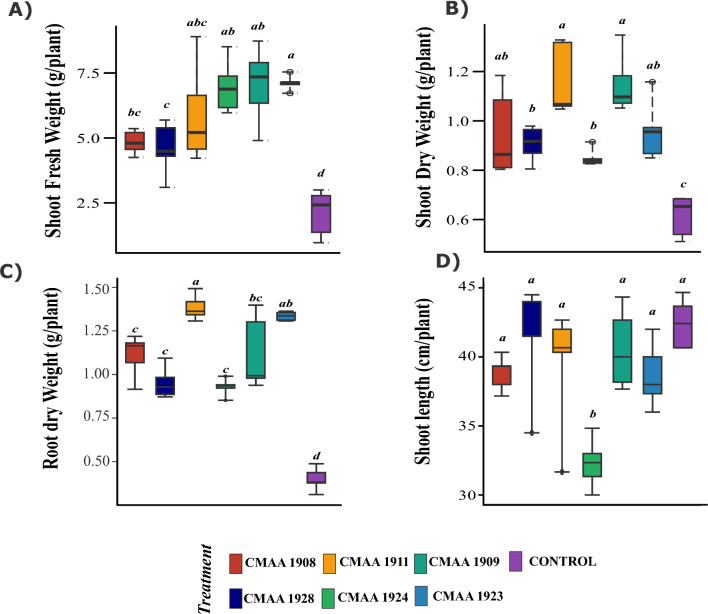
Physiological responses of maize plants to archaeal inoculation. Fresh shoot weight (**A**), Shoot dry weight (**B**), Root dry weight (**C**), and Shoot length (**D**). Error bars indicate the standard error of the mean, and letters above the bars denote significant differences based on ANOVA followed by the post hoc Tukey HSD (*p < *0.05)

The evaluation of archaeal inoculation revealed a significant increase in fresh biomass (Tukey's HSD at 5%) in plants treated with *Haloadaptatus* strains compared to the control. The most notable enhancements were observed in plants treated with the archaea strains CMAA 1923, CMAA 1909, and CMAA 1924 (Fig. 9B). This surge in fresh weight likely corresponds to enhanced water accumulation in response to salt stress. However, non-inoculated plants exhibited more severe symptoms under 250 mM NaCl irrigation, indicating heightened susceptibility to osmotic stress induced by saline irrigation (Fig. 8).

Inoculation with haloarchaea significantly improved plant growth under salt stress. Compared to non-inoculated controls, treated plants exhibited increased root and shoot dry biomass, with strains CMAA 1911 and CMAA 1909 showing the most pronounced effects (Fig. 8). Root development was particularly enhanced: CMAA 1911 and CMAA 1923 achieved the highest root dry weight, surpassing control values by a significant margin (*p < *0.01; Fig. 9B). While other strains (e.g., CMAA 1908, CMAA 1928) also promoted root and shoot biomass, their impacts were less pronounced, though still statistically significant (*p < *0.05; Fig. 9A–B). Notably, CMAA 1923 preferentially stimulated root growth over shoots parts, highlighting its role in belowground stress adaptation (Fig. 9). Despite these improvements, shoot length remained unaffected across all treatments (*p > *0.05; Fig. 9D). Together, these results demonstrate that haloarchaeal inoculation enhances salt stress tolerance in plants, primarily by boosting root biomass and dry matter allocation. Additionally, we observed that archaeal inoculation enabled maize seeds to germinate in a high NaCl concentration of 200 mM (Figure S2).

**Fig. 10:**
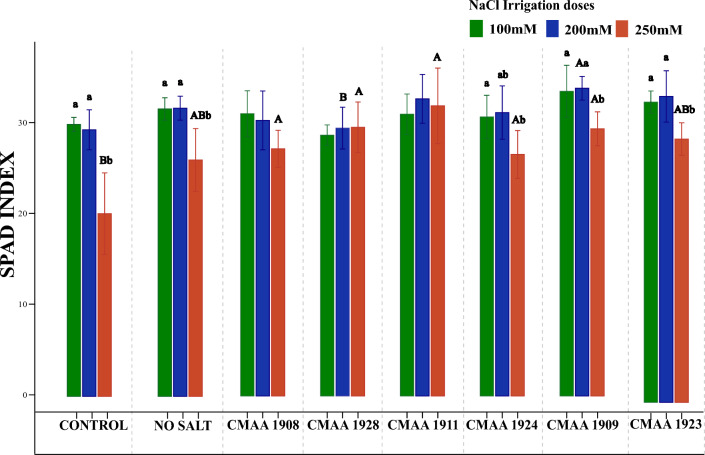
Impact of archaea inoculation on chlorophyll levels in maize plants under saline irrigation. The UPPCASE later represents significant differences within the same treatment across samplings, while the lowercase letter denotes significant differences between all treatments within each sampling. Each color represents the salt concentration in mM during irrigation. Error bars indicate the standard error of the mean, and letters above the bars denote significant differences based on ANOVA followed by the post hoc Tukey HSD (*p < *0.05)

Chlorophyll measurements indicated a decline in the photosynthetic capacity of maize plants corresponding to higher NaCl concentrations in the irrigation water. Nonetheless, inoculated plants displayed an enhanced photosynthetic response and more effective mitigation of salinity effects, as evidenced by their chlorophyll levels (Fig. 10). The most significant effect on Chlorophyll was observed under 250 mM NaCl irrigation. Inoculation with isolates CMAA 1924 and CMAA 1909 resulted in elevated chlorophyll levels in plant leaves. Notably, plants inoculated with strain CMAA 1908 exhibited elevated chlorophyll levels only after the initiation of saline irrigation. The most notable difference in chlorophyll levels emerged in plants irrigated with 250 mM NaCl. Control treatment experienced a reduction of approximately 25% in their initial chlorophyll levels compared to those irrigated without saline after 24 h of irrigation with 250 mM NaCl.

## Unveiling the population dynamics of archaea inoculation in maize rhizosphere under saline irrigation

**Fig. 11:**
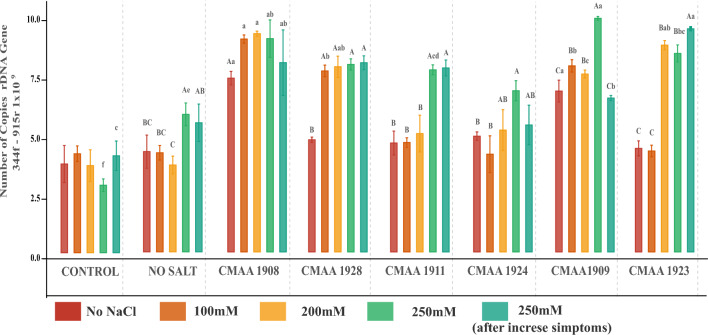
Relative Abundance of archaeal 16 srRNA gene copy number from rhizosphere samples. The UPPCASE later represents significant differences within the same treatment across samplings, while the lowercase letter denotes significant differences between all treatments within each sampling. Each color represents the salt concentration in mM during irrigation. Error bars indicate the standard error of the mean, and letters above the bars denote significant differences based on ANOVA followed by the post hoc Tukey HSD (*p < *0.05)

The qPCR analyses unveiled a direct correlation between the 16S gene copy number in the rhizosphere of inoculated plants and the progressively increasing saline irrigation (Fig. 11), whereas it remained stable in rhizosphere samples from non-inoculated plants. A surge in the number of copies became apparent from the second collection with the initial saline irrigation of 100 mM NaCl. However, significant differences in the archaea population in the rhizosphere of the different treatments demonstrate a distinct colonization potential and density among the strains in the maize rhizosphere. Interestingly, population enrichment was more evident after irrigation treatment with 200 mM NaCl (third sampling). The rhizosphere of plants inoculated with strains CMAA 1908, CMAA 1928, CMAA 1909, and CMAA 1923 displayed the highest copy numbers following irrigation with 200 mM and 250 mM NaCl. However, a decline in copy numbers coincided with seedlings exhibiting symptoms of salt-induced damage, except in treatments with strains CMAA 1928, CMAA 1911, and CMAA 1923 CMAA 1923, which maintained a more stable archaea population even during the period of incrementally increasing salt irrigation (Fig. 11).

### Genome sequencing and annotation of potential mechanisms for plant salt tolerance induction in *Haladaptatus* strains

**Fig. 12:**
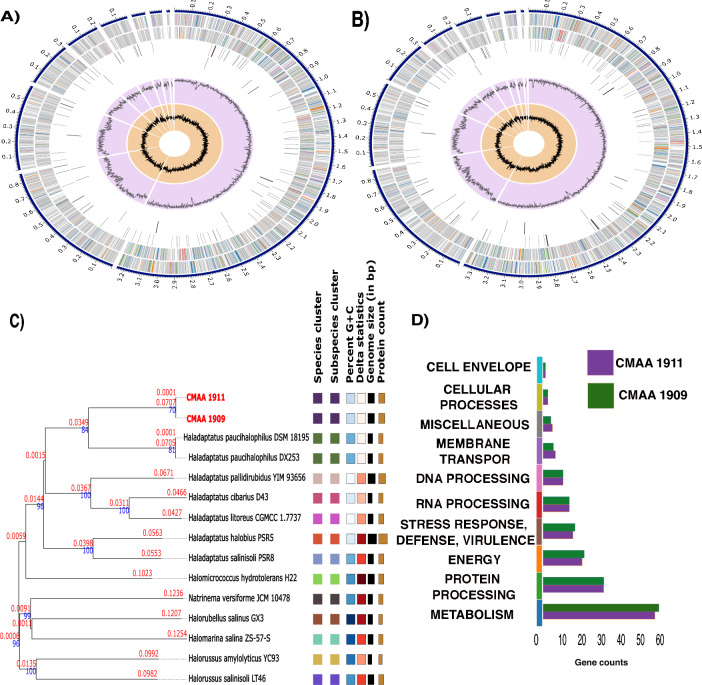
Circular genome view of haloarchaea CMAA 1911 **A** and 1909 **B**. Phylogenetic tree based on the genomes using TYGS, indicating a probable new species of haloarchaea closely related to the genus *Haladaptatus* **C** Functional annotation of the *Haladaptatus* sp. strain's genome. **D** Seed Functional annotation from genome CMAA 1911 (puple) and CMAA 1909 (dark green)

Whole-genome sequence analysis of the strains provided reliable taxonomic classification and identified genes or pathways potentially contributing to the plant response to salinity stress. The strain exhibited the closest relation to the genus *Haladaptatus*, with 87% similarity according to the GBDT. Protein-encoding genes included those implicated in potassium, nitrogen, phosphorus, and iron metabolism, all associated with promoting plant growth. Strains CMAA 1911 and CMAA 1909 harbored genes related to carotenoids, phytoene synthase, and L-tryptophan, directly correlating with phytohormone production. The pairwise ANI similarity between strains CMAA 1911 and CMAA 1909 was 99.95%, with some gene differences, primarily those related to osmotic and oxidative stress. The CMAA 1909 genome contains 246 genes encoding for oxidative stress, while CMAA 1911 has approximately 334 genes. Notably, the CMAA 1911 genome carries a higher number of osmotic stress genes compared to CMAA 1909 (109 and 56, respectively) (Fig. 12D).

Additionally, genes encoding amino acid biosynthesis, such as arginine, cysteine, and methionine, crucial for plant development under salinity stress, were identified. A gene cluster associated with the production of compatible solutes, such as trehalose, betaine, proline, and ectoine, exhibited a strong connection to the mechanisms of uptake, transport, and synthesis. The SEED functional subsystem pinpointed key roles in the strain's potential to induce plant salt tolerance. Strain CMAA 1911 harbored 59 genes associated with plant hormone production via auxin and tryptophan synthase, while strain CMAA 1909 possessed 35 essential metabolic genes known to enhance the root system.

Interestingly, we highlight the main functions identified through annotations using the PGPr_finder software, which are associated with plant growth promotion and health attributes. In general, we observed slight differences in the quantity of some of these attributes. Another noteworthy finding was the annotation of 151 genes for strain CMAA 1909 and 152 genes for strain CMAA 1911 (Table S4), with the exclusive annotation of plant signal indole volatile metabolism for strain CMAA 1911 (Table S4).

**Fig. 13:**
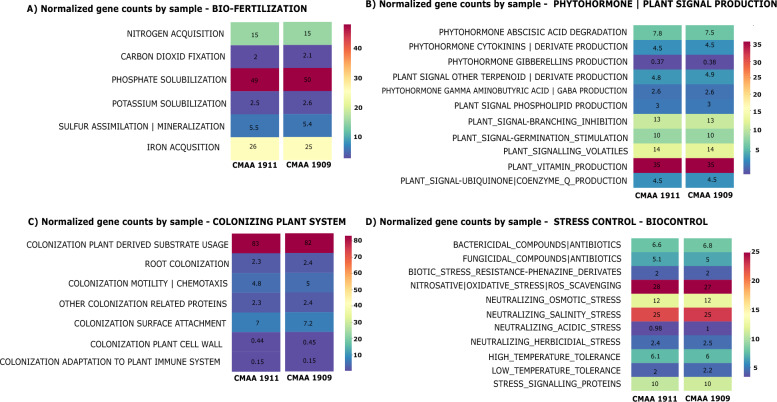
Annotation of genes related to plant growth promotion and stress tolerance induction using PGPG_finder. **A** Normalized gene count associated with biofertilization. **B** Genes involved in phytohormone production. **C** Genes related to plant colonization processes. **D** Genes associated with the induction of tolerance to abiotic and biotic stress

Regarding genes related to biofertilization, the strains exhibited a significant number of genes associated with phosphate solubilization, followed by genes related to iron and nitrogen acquisition (Fig. 13A). For genes related to phytohormones, the function with the highest number of genes was vitamin production and plant signaling. Interestingly, we observed a difference between the isolates in relation to the degradation of the stress hormone abscisic acid (ABA) (Fig. 13B).

Beside, we found genes related to plant colonization, particularly the number of genes associated with substrate usage derived from plant colonization, with 83 genes related to this function. Moreover, we found that strain CMAA 1909 has 7.2 genes related to surface colonization, while strain CMAA 1911 has 7. In the search for potential mechanisms related to plant protection against stress, particularly salinity, we observed that the strains possess more genes related to oxidative, saline, and osmotic stress control (Fig. 13D), possibly responsible for optimizing these strains'responses to stress.

## Discussion

Microorganisms play a crucial role in shaping ecologically sustainable and efficient crop management systems. To devise effective strategies for their optimal utilization in agricultural production, it is imperative to delve into their unique characteristics, including resilience to harsh environments, genetic diversity, and the potential to associations with cultivated plants [17, 48]. Employing microbial bioinoculants in saline soils offers a means to mitigate saline stress, promote plant development, and enhance disease resistance [49, 50].

Harnessing the potential of microorganisms in saline environments, farmers can effectively manage and mitigate the impacts of abiotic stress on crop [51]. They can improve soil health, nutrient uptake, and water utilization, ultimately resulting in increased yield and profitability. Studies have highlighted the promising role of growth-promoting rhizobacteria (PGPR) isolated from halophyte and halotolerant plant species as enhancers of crop growth in saline-affected agricultural areas. [49, 52–55].

In this study, delving into the microbiome of plants acclimatized to saline stress conditions, exemplified by the halophyte plant *A. nummularia*, unveiled a significant potential for bio-protection by microorganisms suitable for agricultural use in salinity conditions. This can be largely attributed to the community's adaptation to osmotic regulation and its ability to survive under these conditions [56].

The metataxonomic approach was employed to investigate the effects of saline irrigation on the rhizosphere microbiome of the halophyte plant *A. nummularia* cultivated in experimental fields in the Brazilian Caatinga biome. The findings revealed a reduction in both diversity and richness of the microbial community in the rhizospheres of plants subjected to saline irrigation. It is important to note that even minimal salt quantities in the soil could suffice to influence the biological community of a given environment [19]. This observation also indicates the recruitment and maintenance of a specialized halophilic microbiome adept at mitigating the adverse effects of saline stress through potential regulatory mechanisms, with osmotic regulation playing a crucial role in adapting to stress levels.

Consequently, recruiting and interacting with a rhizosphere microbiome more specialized to these conditions may assist the development and growth of *Atriplex* under the stress conditions imposed by elevated environmental salt concentrations. Benidire et al., observed that differences in genus composition between environments are mainly associated with salinity's influence on the population size of dominant species and the selection of subdominant taxa that are more specialized or tolerant to increased soil salinity [57]. The abundance analysis revealed the impact of salinity on the taxonomic composition of the *A. nummularia* rhizosphere microbiome. There was an enrichment of the phyla *Proteobacteria (Pseudomonadota)* and *Actinobacteriota*, which commonly dominate in saline soils and semi-arid [58, 59], as well as the predominant archaea from the *Halobacteriaceae* family with halophilic characteristics.

Microorganisms, including bacteria, fungi, protists, and archaea, serve as vital functional components of the plant microbiome and can found in both the rhizosphere and endosphere [18]. Although relatively unexplored, these groups of microorganisms may play a crucial role in promoting plant growth, providing essential nutrients, and protecting against various abiotic stresses [20, 60]. The exclusive presence of halophilic archaea *Haladaptatus* in the rhizosphere of *A. nummularia* cultivated under high salt concentrations underscores its potential role in helping the host mitigate the deleterious effects of salt stress. *Haladaptatus spp.* isolated from saline environments showcases robust adaptive potential to survive adverse environmental conditions [61].

The enrichment of functional categories related to the transport of osmoprotective sugars, such as proline, betaine, glycine, and trehalose, along with the H⁺: K⁺ transport system, suggests that salinity modulates a rhizosphere community with enhanced osmoregulatory potential. Indeed, osmoadaptation mechanisms in halophilic members of the order *Halobacteriales*, particularly *Haladaptatus*, involve the synthesis and absorption of compatible solutes, such as glycine-betaine [62–64].

In addition to investigating the impacts of salinity in modulation the *A. nummularia* microbiome, greenhouse experimental was conducted to assess the potential of *Haladaptatus strains* hosting the *A. nummularia* rhizosphere, in alleviating saline stress in maize. Following the inoculation of the strains into the soil, significant effects were observed in inducing tolerance to saline stress in maize seedlings subjected to irrigation with a gradual increase in NaCl concentration (100 mM, 200 mM, and 250 mM).

It is well understood that elevated salt concentrations in the soil reduce the plant's photosynthetic capacity, inhibiting or diminishing leaf growth and accelerating senescence [56, 65, 66]. However, the inoculated plants exhibited enhanced development despite the deleterious effects of salinity, primarily fostering an increase in root biomass, thereby improving the nutrient acquisition and enhancing stress tolerance. Additionally, leaves of seedlings grown in previously inoculated soil also displayed higher SPAD levels, indicating greater photosynthetic capacity.

The application of plant growth-promoting bacteria (PGPB) involves several knowledge gaps concerning their interaction mechanisms and beneficial effects on plant growth and health, including their ability to colonize the rhizosphere and thrive under varying environmental conditions [66]. Therefore, when developing and applying bioinoculants, it is crucial to consider the capacity of PGPB to successfully compete and colonize the rhizosphere. In this study, although the specific interaction mechanisms remained elusive due to their complexity, the successful colonization capacity of *Haladaptatus* strains in the maize rhizosphere was evidenced by increase of the 16S rRNA gene copy number, as determined by qPCR analyses. The population dynamics varied according to the strain and NaCl concentration of soil. Haloarchaeal groups are known for their adaptability to fluctuations in in external osmolarity [68]. However, the mechanisms shaping the interaction between plants and archaea, as well as their establishment in the rhizosphere environment still remain unknown. The colonization potential of different strains correlated with the gradual increase in saline concentration. Interestingly, plants displaying improved physiological responses and growth were inoculated with strains CMAA 1908 and CMAA 1923 which exhibited a higher population density in the rhizosphere compartment. This correlation suggests a direct contribution to the development of plant-associated functions aimed at mitigating the effects of salts on growth.

Recent studies have shed light on the significant presence of archaea in plant-associated ecosystems, both above- and below-ground phytobiomes. Despite being relatively underexplored and often overlooked in plant microbiomes, their widespread association with plants suggests an as-yet-unknown role in host health [69]. Metagenomic insights indicate the genetic capacity of archaea to interact with plants through (i) promotion of plant growth via auxin biosynthesis, (ii) nutrient supply, and (iii) protection against abiotic stress, especially oxidative and osmotic stress [24]. White et al., reported for the first time the ability of the thermophilic archaea *Sulfolobus acidocaldarius* to secrete the plant growth-promoting hormone indole acetic acid (IAA) at levels a thousand times higher than those observed in plant extracts [70], which points to the possibility of the associated archaeal promoting plant growth. Additionally, the role of phosphate-solubilizing halophilic archaea in supporting the development of plants thriving in hypersaline soils by enhancing phosphorus availability has been proposed [60].

The accumulation of information has sparked scientific interest in investigating the role of archaea in plant health and their potential symbiosis in ecosystems. One mechanism directly related to plant-microorganism interaction is the production of N-acyl-L-homoserine lactones (AHLs), signaling molecules used by *Pseudomonas* species to modulate plant growth and defense responses [71]. As in bacteria, the production of these signaling molecules by archaeal isolates suggests the possibility of signaling and interactions between plants and archaea that modulate plant growth [72]. Song et al. demonstrated, for the first time, the interaction of soil archaea with *Arabidopsis thaliana,* promoting growth and inducing systemic resistance against the necrotrophic bacterium *Pectobacterium carotovorum subsp. Carotovorum* SCC1 and the biotrophic bacterium *Pseudomonas syringae* pv. *tomato* DC3000. *Nitrosocosmicus oleophilus* MY3, an ammonia-oxidizing archaea colonized the root surface of *Arabidopsis* and increased resistance against pathogenic species by emitting volatiles and activating the salicylic acid-independent signaling pathway, mechanisms similar to that found in soil bacteria and fungi [29]. Also, a recent study showcased the ability of the haloarchaea *Halolamina pelagica* to alleviate the drought stress in wheat plants and induced the expression of key stress-responsive genes [27]. The remarkable viability an adaptability of archaea to extreme environments also suggests their potential contribution to plant tolerance against various abiotic stresses, including high salinity, limited water availability, and high temperature. Metagenomic analysis of the rhizosphere of *Jatropha curcas*, a plant adapted to saline and high-temperature conditions, showed a high abundance of *Crenarchaeota* and *Euryarchaeota* [73]. Although the mechanisms are yet to be elucidated, members of these groups may play a role in enhancing tolerance to salt stress and high temperature. Despite the metagenomic evidences, many of these mechanisms remain unknown.

*Haladaptatus* isolates recovered from the *A. nummularia* rhizosphere growing in saline-irrigated fields exhibited normal growth in a medium with 25% NaCl concentrations. Notably, the formation of salt crystals was observed in cultivation areas on agar plates containing 23% NaCl (Fig. 6C). This suggests that the potential to alleviate salt stress in the rhizosphere may be directly linked to mechanisms of salt accumulation in the biofilm, helping to increase root growth and consequently alleviating symptoms of salt stress in inoculated plants. One adaptative strategy observed in halophilic archaea, known as “salt-in,” involves the intracellular accumulation of salts to regulate the osmotic balance with the external environment, typically employing ATP-dependent proton transport pumps [74]. Another mechanism commonly used by halophilic microorganisms is the production of osmoprotectant compounds, such as proline, glycine, betaine, and ectoine [75].

Mainly due to the limitations in culturing archaea, genome mining has proven to be an essential tool for identifying potential genes related to plant stress responses in conjunction with the inoculation of halophilic archaea. The genome analyses of *Haladaptatus* strains revealed a repertoire of genes potentially crucial for enhancing plant resilience to saline stress, particularly those involved in tryptophan biosynthesis and metabolism. Tryptophan metabolism stands out as a pivotal pathway for the production of indole-3-acetic acid (IAA), a well-known phytohormone involved in abiotic stress response which may have indirectly promoted seed germination in the medium containing 200 mM NaCl (Figure S3) [76, 77]. Moreover, the presence of genes related to phytohormones (Fig. 13B), including those contributing to ABA synthesis via the carotenoid pathway, indicates the capacity of *Haladaptatus* strains to modulate plant physiological responses. Additionally, the detection of genes involved in the production of compatible solutes, such as trehalose, betaine, proline, and ectoin, indicates the ability to regulate osmotic balance and protect cellular structures from salt-induced damage. These findings underscore the genetic potential of *Haladaptatus* for enhancing salt tolerance. Future investigations focusing on differential expression analyses of some target genes in the rhizosphere of inoculated plants may validate the mechanisms underlying salt stress mitigation.

This is the first report of *Haladaptatus* sp., an archaeal species, aiding in the mitigating saline stress in plants, unveiling novel insights into the potential agricultural applications of archaea.

## Conclusion

The increase of soil salinity modulates the rhizospheric microbiome of halophyte plant *A. nummularia* growing in experimental fileds subjected to saline irrigation, resulting in an enrichement of halophilic arqueon, especially belong to the *Haloadaptatus* genus. Strains of *Haladaptatus* isolated from the rhizosphere showed potential in ameliorating saline stress in maize seeddlings upon soil inoculation in greesnhouse experiments. Furthermore, *Haladaptatus* strains exhibited competitive viability, with population density increasing in accordance with the gradual rise in salt concentration of irrigation. Genome analysis of *Haladaptatus* unveiled a repertoire of genes supporting the hypothesis that these microorganisms can protect plants from the impacts of soil salinization.

## Supplementary Information

Additional file 1.

Additional file 2.

## Data Availability

Raw amplicon sequencing data is available from NCBI in the bioproject number PRJNA1168574 and through SRA number SRS22852924. The whole genome shotgun project has been deposited at DDBJ/ENA/GenBank under the accession: *Haladaptatus* sp. CMAA 1909-JBIOAV000000000 and *Haladaptatus sp.* CMAA 1911-JBIOAW000000000.
