# Supplementary material for: Harnessing haloarchaea from halophyte Atriplex nummularia rhizosphere to enhance salt stress tolerance in maize seedlings
Source: Environ Microbiome. 2025 Dec 11;21:12. doi: 10.1186/s40793-025-00698-2 (PMC12801921; doi:10.1186/s40793-025-00698-2)
Supplement: Supplementary file 2 — Additional file 2. [file 40793_2025_698_MOESM2_ESM.docx]

| **Determination of Soil Chemical Properties** | | | **C.E** | **pH** | **P** | **K** | **Na** | **Ca** | **Mg** | **H+Al** | **SB** | **CTC** | **V** | **Cu** | **Fe** | **Mn** | **Zn** |
| --- | --- | --- | --- | --- | --- | --- | --- | --- | --- | --- | --- | --- | --- | --- | --- | --- | --- |
|  |  |  | **(dS/m)** |  | **(mg.kg⁻¹)** | **(cmol.kg⁻¹)** | | | | | | | **%** | **(mg.kg⁻¹)** | | | |
| CEC | Rhizosphere | P1 | 5.76 | 7.8 | 43.2 | 1.5 | 1.74 | 2.6 | 1.1 | 0 | 6.9 | 6.9 | 100 | 0.78 | 36.1 | 48.91 | 4.96 |
|  |  |  |  |  |  |  |  |  |  |  |  |  |  |  |  |  |  |
| CEC | Rhizosphere | P2 | 5.55 | 8.1 | 63.51 | 2.07 | 1.92 | 2.3 | 1.2 | 0 | 7.5 | 7.5 | 100 | 0.79 | 37.82 | 48.06 | 7.07 |
| CEC | Rhizosphere | P3 | 5.98 | 8.2 | 33.69 | 1.65 | 1.95 | 2.0 | 0.8 | 0 | 6.4 | 6.4 | 100 | 0.82 | 39.28 | 43.37 | 2.46 |
|  |  |  |  |  |  |  |  |  |  |  |  |  |  |  |  |  |  |
| CEC | Bulk Soil | P1 | 6.51 | 8.1 | 77.33 | 2.82 | 2.58 | 4.6 | 1.6 | 0 | 11.6 | 11.6 | 100 | 0.87 | 43.54 | 68.56 | 4.77 |
|  |  |  |  |  |  |  |  |  |  |  |  |  |  |  |  |  |  |
| SNT | Rhizosphere | P1 | 3.6 | 7.3 | 118.6 | 0.19 | 0.26 | 4.7 | 1.5 | 0 | 6.7 | 6.7 | 100 | 0.78 | 34.52 | 70.23 | 7.90 |
| SNT | Rhizosphere | P2 | 1.43 | 7.0 | 29.59 | 0.20 | 0.16 | 2.2 | 1.1 | 0.2 | 3.7 | 3.9 | 93.8 | 0.70 | 32.22 | 48.76 | 3.78 |
| SNT | Rhizosphere | P3 | 1.87 | 6.8 | 48.45 | 0.25 | 0.11 | 2.8 | 1.4 | 0.2 | 4.6 | 4.8 | 95 | 0.69 | 40.34 | 64.09 | 7.76 |
|  |  |  |  |  |  |  |  |  |  |  |  |  |  |  |  |  |  |
| SNT | Bulk Soil | P1 | 2.77 | 8.0 | 97.21 | 0.84 | 0.75 | 3.7 | 1.5 | 0 | 6.8 | 6.8 | 100 | 0.59 | 38.54 | 58.65 | 4.86 |
|  |  |  |  |  |  |  |  |  |  |  |  |  |  |  |  |  |  |

**Table S1** – Caatinga Soil characterization

Soil chemical properties: pH was measured using 0.01 M CaCl2; H+Al content was measured using the SMP-buffer method; P, K, Ca, and Mg content were measured using resin extraction; Na content was measured using Al acetate at pH 7; SB = Sum of bases; CEC = Cation exchange capacity; Al content was measured using potassium chloride extraction; S content was measured using calcium phosphate extraction; B content was measured using hot water extraction; Cu, Fe, Mn, Zn, content were measured using DTPA extraction; EC = Electrical conductivity was measured using a 1:1 water extraction; N content was measured using the Kjeldahl method.

**Table S2 – Soil pot experimental characterization**

| **Properties** | **Unitis** | **Value** |
| --- | --- | --- |
| ***Soil physical properties*** | | |
| Total sand | g/kg | 429 |
| Clay |  | 528 |
| Silt |  | 43 |
| ***Soil chemical properties*** | | |
| OM | g.dm-3 | 19 |
| C.E | dS/m | 0,18 |
| pH |  | 4,5 |
| P | mg.kg⁻¹ | 5 |
| K | cmol.kg⁻¹ | 0,85 |
| Na |  | - |
| Ca | cmol.kg⁻¹ | 12,3 |
| Mg |  | 9,75 |
| H+Al |  | 27 |
| SB |  | 23 |
| CTC |  | 50,23 |
| V | % | 46,25 |
| Cu | mg.kg⁻¹ | 1 |
| Fe |  | 76 |
| Mn |  | 1,7 |
| Zn |  | 0,7 |

Soil chemical properties - OM = Organic matter. pH was measured using 0.01 M CaCl2; H+Al content was measured using the SMP-buffer method; P, K, Ca, and Mg content were measured using resin extraction; Na content was measured using Al acetate at pH 7; SB = Sum of bases; CEC = Cation exchange capacity; Al content was measured using potassium chloride extraction; S content was measured using calcium phosphate extraction; B content was measured using hot water extraction; Cu, Fe, Mn, Zn, Cl, Cd, Cr, Pb, and Ni content were measured using DTPA extraction; EC = Electrical conductivity was measured using a 1:1 water extraction; N content was measured using the Kjeldahl method. Soil physical properties – Soil density was obtained by deformed sample; soil moisture was obtained with two methods: field capacity (FC) and permanent wilting point (PWP); granulometry measurement was obtained by pipette method (Clay < 0.002 mm, silt = 0.053 – 0.002 mm, and total sand = 2.00 – 0.053 mm).

**Table S3.** Taxonomic identification based on the 16S rRNA gene of archaea against the Eztaxon database.

| **Strain** | **Top-hit taxon** | | **Similarity (%)** |  | | **Top-hit taxonomy** | |
| --- | --- | --- | --- | --- | --- | --- | --- |
| **CMAA 1923** | **DX253** | | **95.72** |  | Archaea;Euryarchaeota;Halobacteria;Halobacteriales;Halobacteriaceae;Haladaptatus | |  |
| **CMAA**  **1909** | | **DX253** | **96.67** |  | Archaea;Euryarchaeota;Halobacteria;Halobacteriales;Halobacteriaceae;Haladaptatus | |  |
| **CMAA 1911** | **DX253** | | **93.61** |  | Archaea;Euryarchaeota;Halobacteria;Halobacteriales;Halobacteriaceae;Haladaptatus | |  |
| **CMAA 1928** | **DX253** | | **96.32** |  | Archaea;Euryarchaeota;Halobacteria;Halobacteriales;Halobacteriaceae;Haladaptatus | |  |
| **CMAA 1908** | **DX253** | | **97.49** |  | Archaea;Euryarchaeota;Halobacteria;Halobacteriales;Halobacteriaceae;Haladaptatus | |  |
| **CMAA 1924** | **DX253** | | **97.89** |  | Archaea;Euryarchaeota;Halobacteria;Halobacteriales;Halobacteriaceae;Haladaptatus | |  |

**FIGURE S1**

**
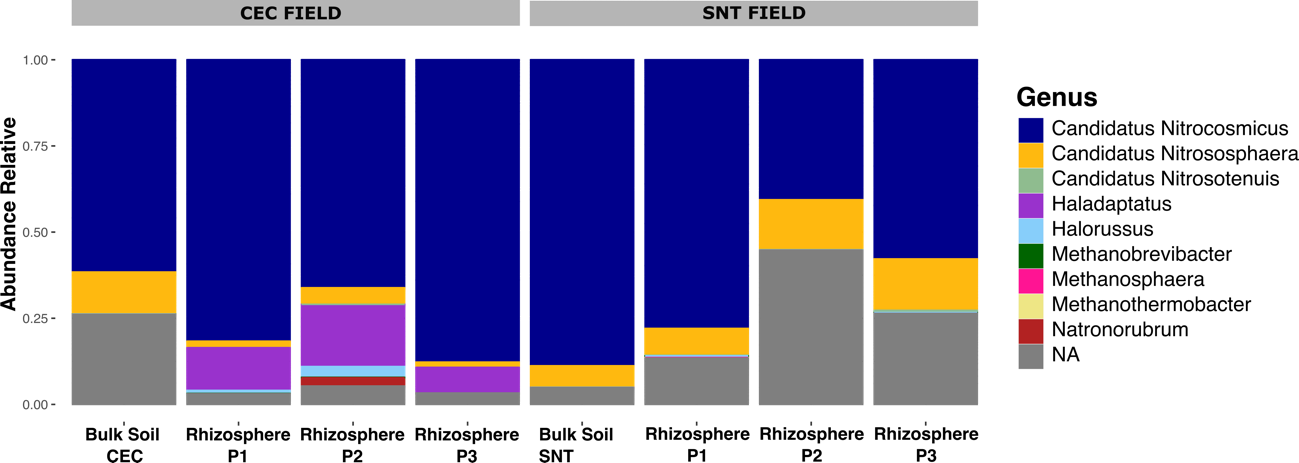
**

**FIGURE S1 –** Relative abundance of Archaeal ASVs at the genus level

**
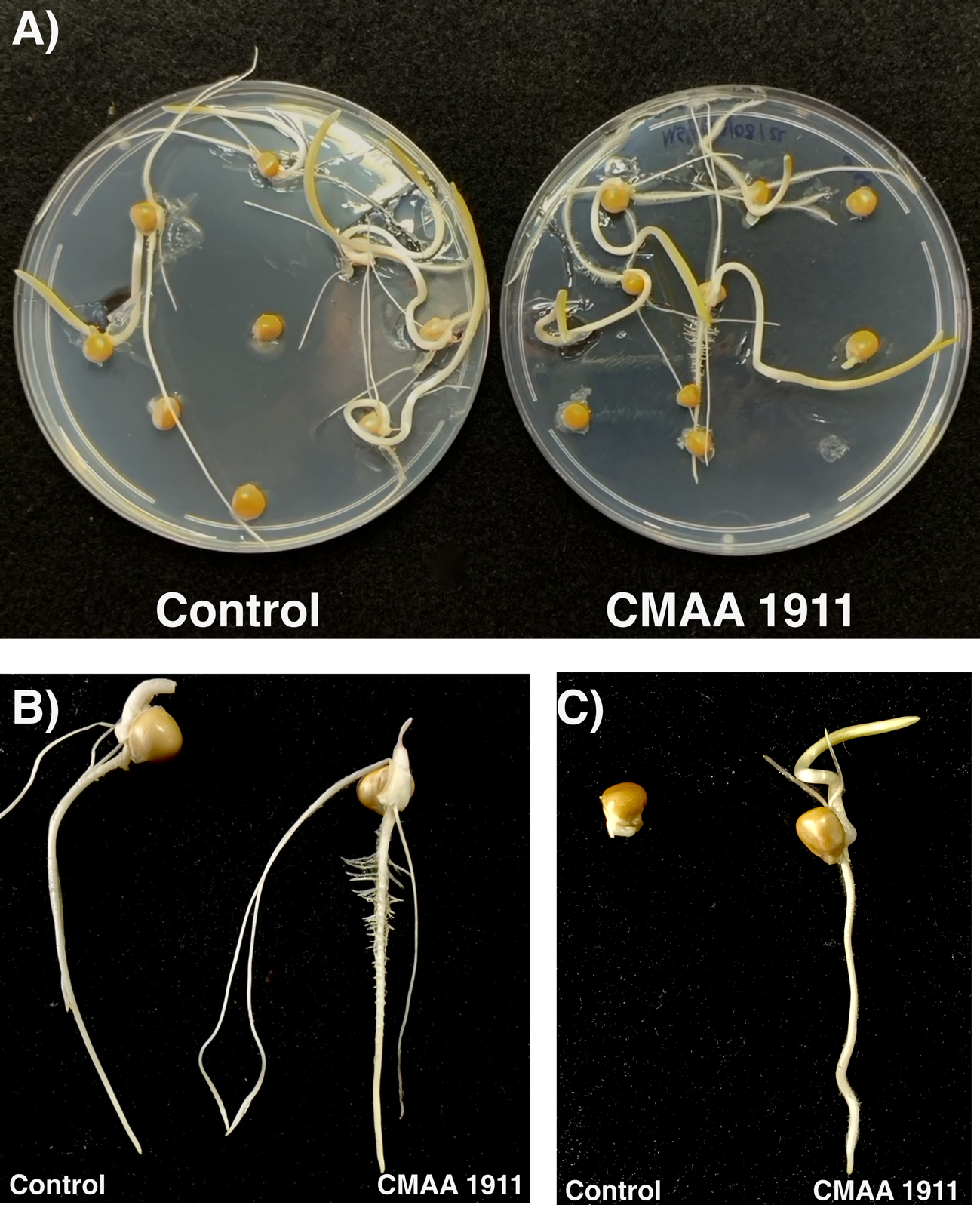
FIGURE S2**

**FIGURE S2 –** Seed germination test with archaeal lineages. (A) Murashige and Skoog (MS) culture medium without NaCl. (B) Germinated seeds in MS medium without NaCl. (C) Culture medium with the addition of 200 mM NaCl. The control treatment refers to seeds that were not inoculated with the archaeal strain CMAA 1911. The CMAA 1911 treatment refers to the inoculation of the halophilic archaeon *Haladaptatus* sp.
